# Supplementary material for: A Global Screen for Assembly State Changes of the Mitotic Proteome by SEC-SWATH-MS
Source: Cell Syst. 2020 Feb 26;10(2):133–155.e6. doi: 10.1016/j.cels.2020.01.001 (PMC7042714; doi:10.1016/j.cels.2020.01.001)

Q8WU90 | ZC3HF_HUMAN | ZC3H15 DFRP1 LEREPO4 HSPC303 HT010 MSTP012 PP730
Monomer MW [kDa]: 48.602 Monomer expected elution fraction: 46

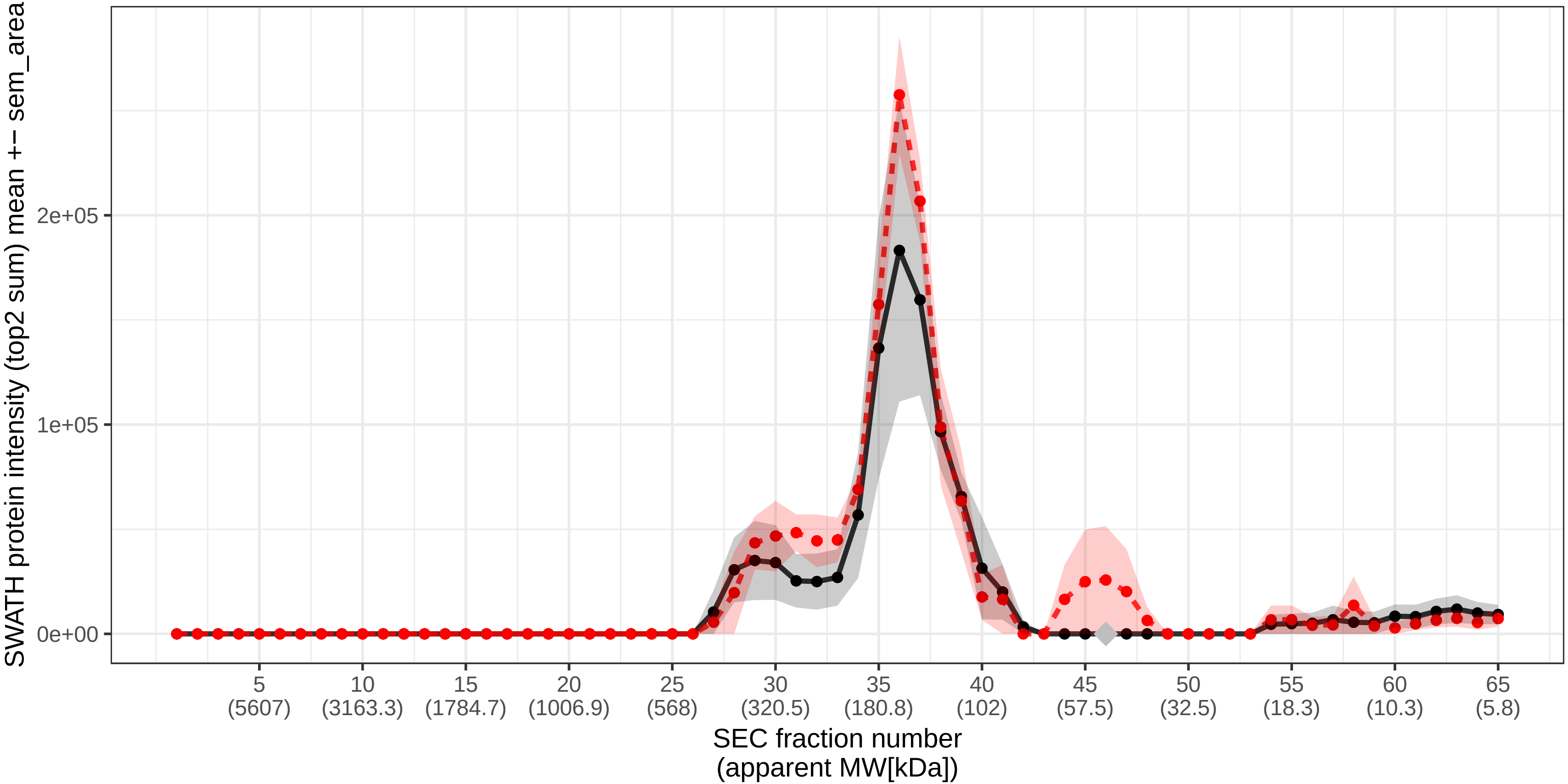

Supplement: Data S1. SEC-SWATH-MS Protein Chromatograms, Related to Figure 1 [file mmc6.zip › SECchrom_Q8WU90_ZC3HF_HUMAN_ZC3H15_DFRP1_LEREPO4_HSPC303_HT.pdf]
